# Note from Dr. Copland to the Editor

**Published:** 1820-12

**Authors:** 


### *Note from* Dr. Copland *to the* Editor.

*2, Terrace, Walworth; Nov.* 15, 1820.

SIR,

THE papers of Dr. Pierson and Mr. Down, on the relative quantities of oxygen gas consumed during respiration, in high or low states of atmospheric temperature, as well as your own observations upon the subject, in the very excellent *Proëmium* to the 44th volume of the London Medical Journal, did not fall in my way until a few days since.

I now take the earliest opportunity in my power to inform you, that the doctrines stated by those gentlemen, although originating with them both, yet did not *first* originate with either. Before, however, I proceed to point out to you my own claims as being the first proposer of those doctrines, I will most readily allow an equal share of originality to them; and consider it as a very favourable proof of the correctness of our opinions, that *three* gentlemen entertained them, independently of each other.

The experiments of Crawford, Lavoisier, Sequin, and Fyfe, and a few made by myself, were the data from which, in 1815, I drew my conclusions; and at that time they were the only ones (so far as I know) which had clearly shown that different degrees of temperature and states of the system modified the products of respired air. My friend Dr. Fyfe gave a detail of his experiments on this subject in the year 1814. Towards the close of the same year, my attention was directed to this subject. The experiments then made by me were directed principally to the effects of temperature in modifying the results. At the same time they confirmed, in my opinion, the inferences of Ellis, Allen, and Pepys, that no oxygen passed into the blood, but that carbon was evolved. In the following spring, while engaged in the preparation of my prescribed dissertations, previously to the acquisition of a degree in medicine from the University of Edinburgh, I gave in one of them* a short sketch of my opinions deduced from these experiments; and endeavoured to show the *modus operandi* of heat and moisture in favouring the production of fever and diseases implicating the biliary functions. Not long afterwards I took the opportunity, which an appointment in a tropical country afforded me, of repeating those experiments under a vertical sun, and in a highly insalubrious climate, which were before performed in an artificial one.

I expect soon to lay before the public the opinions deduced from them, which have been confirmed by the experience and observation I have been enabled to make in that climate. I may however remark, that the vicarious operations of the lungs, liver, and skin, (the skin very remarkably in negroes,) which my previously-formed opinions led me to look for, were to me satisfactorily confirmed.

In order that I may give you, for insertion in a future Number of your Journal, the dissertation in question, I have written to Professor Duncan to endeavour to procure it.

* A Commentary on the 24th Aph. of the 4th Sect. of the Aphorisms of Hippocrates.

